# SBAR checklist for the transition of care in nursing in the Pediatric Intensive Care Unit

**DOI:** 10.1590/0034-7167-2025-0398

**Published:** 2026-10-19

**Authors:** Thales Henrique Santos, Juliana Teixeira Jales Menescal Pinto, Micheline Veras de Moura, Izaura Luzia Silvério Freire, Fernanda Maria Wanderley Cavalcanti Freire, José Segundo Barbosa, Sandra Ávila Cavalcante, Jayana Teixeira Jales Menescal Pinto

**Affiliations:** I Universidade Federal do Rio Grande do Norte. Natal, Rio Grande do Norte, Brazil; II Universidade do Porto, Escola Superior de Enfermagem do Porto. Porto, Portugal

**Keywords:** Communication, Transitional Care, Pediatric Nursing, Intensive Care Units, Pediatric, Nursing Methodology Research., Comunicación, Cuidado de Transición, Enfermería Pediátrica, Unidades de Cuidado Intensivo Pediátrico, Investigación Metodológica en Enfermería.

## Abstract

**Objective:**

To build and validate an instrument based on the SBAR mnemonic for the transition of care in Pediatric Intensive Care Units.

**Method:**

A methodological study based on Pasquali’s framework, developed in three poles (theoretical, empirical, and analytical). Items were drafted via a scoping review (PRISMA-ScR). The instrument underwent content and face validation by 15 experts and semantic analysis by three nursing residents. Agreement was estimated using the CVI and Kappa coefficient (> 0.70).

**Results:**

The final version was consolidated into five domains (Identification, Situation, Background, Assessment, and Recommendation) and 41 items. All items presented CVI and Kappa values above 0.70. Semantic analysis confirmed adequate understanding without the need for further adjustments.

**Conclusion:**

The instrument proved to be valid and applicable, constituting an assistive technology capable of standardizing communication and increasing pediatric patient safety during the handover of information.

## RESUMEN

**Objetivo:**

Construir y validar un instrumento basado en el mnemónico SBAR para la transición del cuidado en Unidades de Cuidados Intensivos Pediátricos.

**Método:**

Estudio metodológico fundamentado en el referencial de Pasquali, desarrollado en tres polos (teórico, empírico y analítico). Los ítems se elaboraron mediante una revisión de alcance (PRISMA-ScR). El instrumento fue sometido a validación de contenido y apariencia por 15 expertos y a un análisis semántico por tres residentes de enfermería. La concordancia se estimó mediante el IVC y el coeficiente Kappa (>0,70).

**Resultados:**

La versión final se consolidó en cinco dominios (Identificación, Situación, Antecedentes, Evaluación y Recomendación) y 41 ítems. Todos presentaron IVC y Kappa superiores a 0,70. El análisis semántico confirmó una comprensión adecuada sin necesidad de ajustes.

**Conclusión:**

El instrumento demostró ser válido y aplicable, constituyendo una tecnología asistencial capaz de estandarizar la comunicación y elevar la seguridad del paciente pediátrico durante el traspaso de información.

## INTRODUCTION

Communication is foundational to health care. It is a dynamic process that encompasses verbal and nonverbal cues, including facial expressions, gestures, and posture ^(1)^. Over the years, the concept of communication has shifted from the simple transfer of information to a complex, interactive process shaped by technological advances and rising expectations for quality and safety in care delivery ^(2)^. In hospital settings, ensuring effective communication among health care professionals remains a critical challenge for promoting patient safety, strengthening relationships with patients and families, and preventing incidents ^(1)^. Brazil’s Ministry of Health recognizes this importance by issuing regulatory measures such as Ordinance No. 1,820/2009, which ensures patients’ right to clear, understandable information about their health status, and Ordinance No. 529/2013, which established the National Patient Safety Program (PNSP), whose goals include “improving communication among health care professionals” ^(3)^. Multiple national and international studies identify ineffective communication as a contributing factor to adverse events (AEs), which can cause physical and emotional harm to patients, prolong hospital stays, and increase health care costs ^(4)^. In a study of Danish hospitals, 86% of communication failures occurred during care handoffs, a critical moment when information must be conveyed clearly and completely between shifts, units, or institutions ^(4)^. In the Pediatric Intensive Care Unit (PICU), communication becomes even more critical given children’s clinical vulnerability and the need for ongoing coordination between the interprofessional team and families. Care handoff, understood as the transfer of responsibility for and information about a patient’s care, should occur in a structured, clear, and safe manner to prevent gaps in care, especially in high-acuity settings ^(5,6)^. From this perspective, the SBAR mnemonic (Situation, Background, Assessment, Recommendation) stands out as a structured communication framework that organizes clinical communication into four essential components, helping ensure that information is conveyed in a concise, standardized, and safe manner. Supported by international organizations such as the Institute for Healthcare Improvement (IHI), SBAR has been increasingly incorporated into nursing practice as a clinical tool, particularly in critical care settings such as the PICU, where communication errors can seriously compromise patient safety ^(7)^. Clinical nursing practice in a university hospital PICU revealed recurrent communication failures, particularly during change-of-shift handoffs, transfers of care, medical record documentation, and physician orders. These failures jeopardize continuity of care, children’s safety, and the quality of the services provided. Taken together, these observations and the paucity of standardized SBAR-based tools for pediatric care handoffs highlight a significant gap in both the literature and clinical practice, underscoring the need to develop and validate tools that structure communication and reduce risk. Accordingly, this study aimed to develop and validate an SBAR-based instrument to support care handoffs for children and adolescents in a Pediatric Intensive Care Unit, strengthening communication among health care professionals, patient safety, and the effectiveness of nursing care.

**Relevance of the study:** This article is a product of the Master’s Thesis^(8)^ entitled “Construction and validation of an instrument for the transition of care for children and adolescents in a Pediatric Intensive Care Unit”, deposited in the institutional repository of the Federal University of Rio Grande do Norte (UFRN) and available at: https://repositorio.ufrn.br/items/db82a1fa-502d-49ca-9ce8-2bb40527e39e.

## OBJECTIVE

To develop and validate an SBAR-based instrument to support nursing care handoffs for children and adolescents in a Pediatric Intensive Care Unit, to improve communication among health care professionals, supporting patient safety, and promoting effective nursing care.

## METHODS

### Ethical aspects

The study was conducted in accordance with national and international ethical guidelines and was approved by the Research Ethics Committee of the Federal University of Rio Grande do Norte (UFRN).

### Study design, period, and setting

This is an original study, methodological in nature and with a quantitative approach. It followed the methodological guidelines recommended for research on the construction and validation of instruments, in accordance with the COnsensus-based Standards for the selection of health Measurement INstruments (COSMIN) checklist from the EQUATOR network and PRISMA-ScR for the review stage^(9,10)^. To ensure transparency and reproducibility of the process, a scoping review was conducted as an initial step to provide the foundation for the construction of the instrument, following the PRISMA-ScR (Scoping Reviews) recommendations. Its protocol was previously registered on the Open Science Framework (OSF) platform (DOI: 10.17605/OSF.IO/D93JM) and is available in open access at the following link: https://osf.io/d93jm/. The study was conducted between 2023 and 2025, in the city of Natal, state of Rio Grande do Norte, Brazil.

### Population or sample; inclusion and exclusion criteria

For the instrument’s theoretical validation phase, the sample comprised 15 expert judges recruited through purposive sampling, including pediatric nurse specialists and faculty members with experience in methodological research and pediatrics. Experts with documented experience and practice in the field were included. Judges who were on vacation, away from work, or on leave were excluded.

### Study protocol

The study was developed in three main poles, according to Pasquali’s framework: Pole 1 - Theoretical Procedures (Grounding and Construction): A scoping review was conducted to identify relevant items, followed by the development of the pilot instrument structured into five domains based on the SBAR. Pole 2 - Empirical Procedures (Validation and Semantics): Evaluation of the instrument by 15 expert judges for content and face validation, in addition to semantic analysis through the brainstorming technique with nursing residents. Pole 3 - Analytical Procedures (Statistical Analysis): Processing and analysis of the collected data to verify agreement indices (CVI and Kappa), ensuring the reliability of the final 41-item instrument.

### Data analysis and statistics

The collected data were organized into electronic spreadsheets and analyzed using descriptive statistics (absolute and relative frequencies, mean, and standard deviation). Content validity was verified using the Content Validity Index (CVI), calculated by the sum of “3” (relevant) and “4” (very relevant) scores divided by the total number of judges. Inter-rater agreement was measured using Cohen’s Kappa coefficient (k). A cutoff point of > 0.70 was adopted to consider the item valid and understandable, according to the adopted methodological framework.

## RESULTS

The instrument, supported by a scoping review, comprised five essential domains: patient identity; current situation; clinical background; assessment of systems status; and recommendations for continuity of care. The first version, consisting of 38 items, was evaluated by 15 experts, whose suggestions were incorporated to enhance the instrument’s clarity and comprehensiveness^(8,11)^. The expert panel was predominantly female (93.33%) and most commonly held a graduate certificate (46.67%) or a master’s degree (40%). Most experts self-identified as Brown (pardo) (60%), were Catholic (66.67%), and lived in Natal (86.67%). Although 86.67% reported no prior SBAR-specific training, this did not affect content validation, which focused on the items’ relevance and representativeness. Mean age was 40.67 years (SD = 7.45), and participants had extensive experience in nursing (mean: 16.8 years) and pediatrics (mean: 11.07 years). Thus, validation consisted of experienced professionals assessing literature-derived indicators for care handoffs supported by structured communication. Table 1 presents the profile of the expert judges who participated in instrument validation.

**Table 1:** Profile of the expert judges who participated in instrument validation. Natal, Rio Grande do Norte, Brazil, 2024

Characteristic	Absolute frequency	%
**Sex**		
Female	14	93.33
Male	1	6.67
**Educational attainment**		
Graduate/residency	7	46.67
Master’s degree	6	40.00
Doctoral degree	2	13.33
**Race/ethnicity**		
Brown (pardo)	9	60.00
White	6	40.00
**Religion**		
Catholic	10	66.67
Protestant	3	20.00
No religious affiliation	1	6.67
Other	1	6.67
**Marital status**		
Married/common-law marriage	10	66.67
Never married	4	26.67
Divorced	1	6.67
**City of residence**		
Natal	13	86.67
Mossoró	1	6.67
Parnamirim	1	6.67
**SBAR-related training**		
Yes	2	13.33
No	13	86.67
**Total**	15	100.00

The evaluation by the experts resulted in modifications to the instrument’s domains, aiming to enhance its clarity and clinical applicability. In the “Identification” domain, the language was adjusted for greater objectivity. In the “Situation” domain, items were simplified to facilitate quick completion. The “Background” and “Assessment” domains were revised to incorporate specific pediatric clinical indicators, excluding redundant items. Finally, the “Recommendation” domain was adjusted to ensure safe continuity of care. Table 2 details the quantitative changes in each domain.

**Table 2:** Quantitative distribution of items by domain before and after expert validation, Natal, Rio Grande do Norte, Brazil, 2025

Domain	Original items	Excluded items	Reformulated items	Included items	Final items
1. Identification	8	0	2	2	10
2. Situation	10	1	4	2	11
3. Background	7	1	3	2	8
4. Assessment	9	2	4	2	9
5. Recommendation	4	1	2	0	3
Total	38	5	15	8	41

It is observed that the final version was consolidated into 41 items, resulting from the inclusion of new questions considered essential, which balanced the exclusion of redundant items. The Content Validity Indices (CVI) were calculated for each item, demonstrating high agreement among the evaluators. Table 3 presents the CVI per domain.

**Table 3:** Average Content Validity Index (CVI) and Average Kappa per domain, Natal, Rio Grande do Norte, Brazil, 2024

Domain	Number of final items	Average CVI	Average Kappa
1. Identification	10	1.00	1.00
2. Situation	11	1.00	1.00
3. Background	8	0.93	0.73
4. Assessment	9	1.00	1.00
5. Recommendation	3	1.00	1.00

**Chart 1:** General recommendations for the instrument’s format, Natal, Rio Grande do Norte, Brazil, 2024

Aspect	Recommendation	Rationale
Paper type	Opaque, sturdy paper	Improved durability and handling
Font size	A4	Facilitates printing and filing
Font	Arial, 12-point	Comfortable reading
Organization	Clear division into domains	Easier navigation through the instrument
Spacing	Space for notes	Enables additional comments

After incorporating the suggested revisions, the values reported above indicate that the instrument provides adequate validity evidence for clinical use. In addition to item-level changes, the expert judges recommended formatting refinements to improve usability for clinicians, including: Printing the instrument on sturdy, opaque paper and using A4 size to facilitate handling. Using Arial, 12-point font to improve readability. Adding clear instructions in the header of each domain. Allowing adequate space for additional notes. These recommendations are intended to ensure that the instrument is practical and accessible for hospital-based health care professionals. Table 4 presents the general recommendations for the instrument’s format.

The finalized instrument contains 41 items distributed across five domains. Figure 1 displays the final validated version.

**Figure 1:**
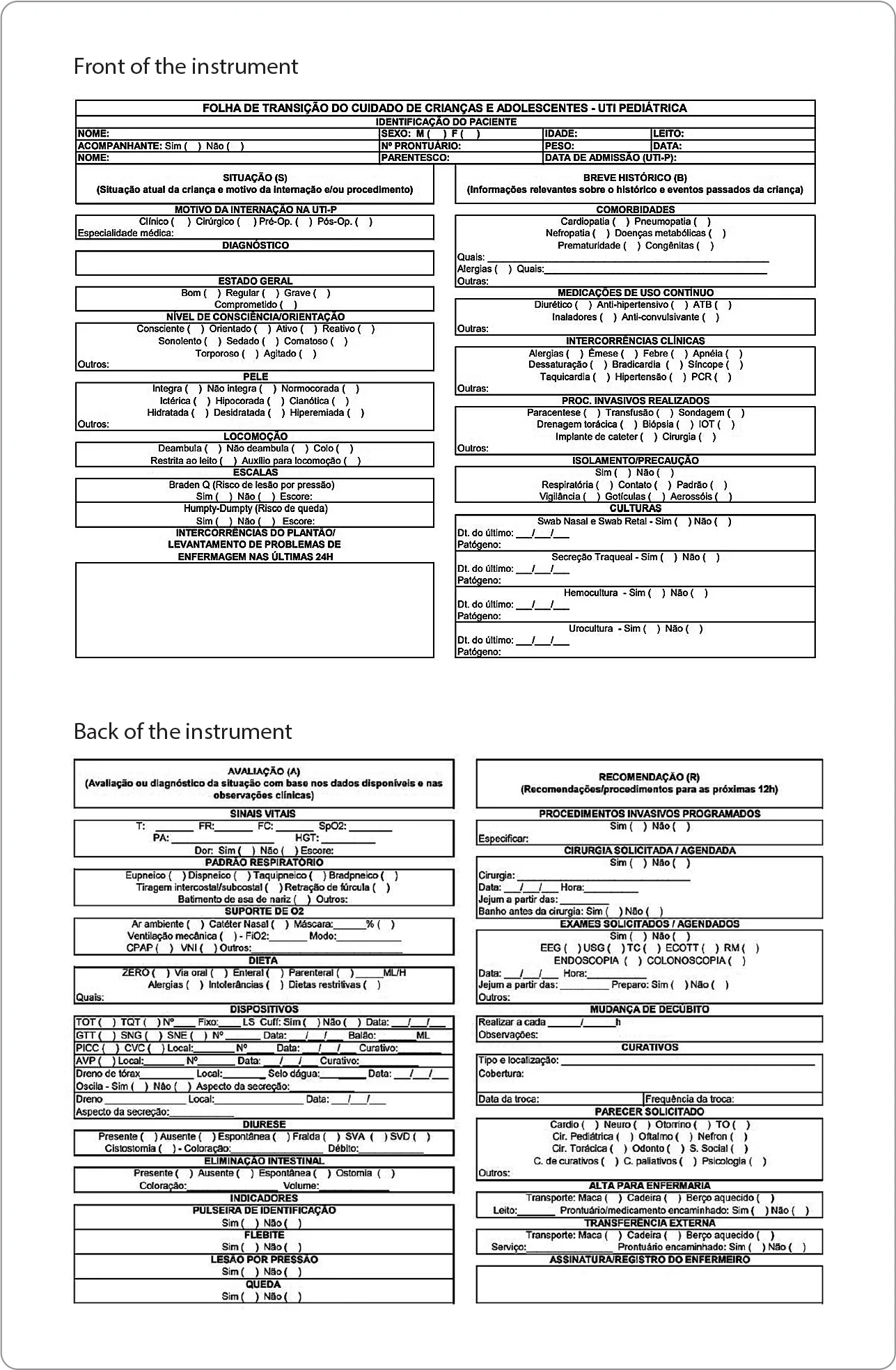
Final version of the instrument (front). Natal, Rio Grande do Norte, Brazil, 2024

## DISCUSSION

Effective communication during change-of-shift handoffs is essential to patient safety in PICUs. Studies have shown that breakdowns in this process can lead to medical errors and adverse events. Frequent interruptions, equipment alarms/noise, and side conversations can undermine the accurate transfer of information among health care professionals ^(12)^. In addition, the lack of standardized communication during shift handoffs can lead to omissions or distortions of clinically significant information about patients’ status. The use of structured tools such as SBAR has been recommended to improve the clarity and concision of the information conveyed, thereby reducing errors ^(13)^. These considerations support the need to validate pediatric-specific instruments that organize and standardize handoff information, promoting safe and effective communication among members of the health care team. Recognizing these challenges and seeking strategies to strengthen handoff communication motivated this study, supporting improvements in quality of care in the PICU. These benefits stem primarily from standardizing language and structuring clinical information-factors that reduce distractions and support more efficient decision making^(14,15)^. Domain 1: Identification: The main change in the “Identification” domain was the addition of the patient’s sex and the caregiver’s relationship to the child. This revision improves record accuracy and supports more individualized care, since the caregiver’s relationship can directly influence the support provided and adherence to the care plan recommended by the health care team ^(16)^. Complete and accurate documentation is essential to care continuity and patient safety. In addition, clearly identifying the accompanying caregiver promotes accountability and supports the family’s active involvement in the therapeutic process-particularly relevant in pediatric settings ^(17,18)^. Domain 2: Situation: In this domain, the accepted suggestions included refining the description of overall condition by classifying it as “fair,” “serious,” or “critical,” providing a more objective and standardized picture of the patient’s clinical status. Broader categories were also added for level of consciousness (including “sedated” and “comatose”), along with age-appropriate parameters for young children, supporting more accurate assessment across age groups. Under “Skin,” the options “hydrated/dehydrated” and “erythematous,” as well as space to document lesions, were added. Collectively, these revisions support a more standardized assessment and facilitate communication among professionals. Studies underscore that standardizing the communication of clinical information is essential to patient safety ^(17)^. Accordingly, incorporating objective descriptors promotes a shared language among professionals and reduces subjectivity in clinical interpretation, which is particularly critical in high-acuity settings such as the PICU ^(19)^.

Domain 3: Brief history: Revisions to the comorbidities section removed the fixed list of conditions and replaced it with broader categories (e.g., renal disease, pulmonary disease, and metabolic disorders), with space for specification. Fields were also added to document allergies and prematurity. These changes make the instrument more flexible while ensuring that clinically relevant information is consistently captured. As such, they reinforce that effective communication of this information is essential to continuity of care and the prevention of adverse events ^(20)^. These revisions align with recent literature recommending more dynamic clinical instruments that can accommodate diverse health conditions and each patient’s individual needs ^(21,22)^. Domain 4: Assessment: The main revisions in this section included refining the “Vital signs” item to emphasize the relevance of point-of-care capillary blood glucose testing for selected patients. Under ventilatory support, a field was added to record ventilator mode, in addition to FiO₂, to better document mechanical ventilation settings. The “Devices” section was expanded to capture details on dressings/wound coverings and central venous access characteristics, providing a more complete picture for the care team. Reliably communicating these details is critical to patient safety, particularly in critical care units ^(23)^. Including specific information on mechanical ventilation and devices can reduce risks associated with care discontinuities and strengthen the team’s ability to respond promptly to clinical changes ^(15)^.

Domain 5: Recommendations: In this domain, the instrument’s layout was reorganized to consolidate “Scheduled invasive procedures” and “Requested/scheduled surgery,” and to redesign the “Requested tests” item for clearer documentation. Space was also added to record the mode of transport used at discharge or during transfer to another facility, and the “Consult requested” item was expanded to include pending consults and psychology consults, supporting continuity and safety of care. Standardized communication tools such as SBAR have been associated with meaningful improvements in handoff quality and clearer delineation of care responsibilities. These refinements allow upcoming actions to be viewed in an integrated way, facilitating care planning by the entire interdisciplinary team^(24)^. Importance of SBAR: Implementing these revisions underscores SBAR’s usefulness for interprofessional communication. Standardizing information transfer supports safer shift handoffs, reduces errors, and helps ensure that all clinically relevant aspects of care are addressed. In turn, refining the instrument strengthens patient safety and the quality of care delivered in the PICU. Validating clinical instruments is essential to ensure the reliability and applicability of tools used in health care practice ^(25)^. Recent studies support combining content and face validation with semantic analysis when developing instruments for clinical use. In pediatrics, validation studies point to a growing body of research using robust methods, with the potential to strengthen practice through methodological innovations in the nursing process. In the present validation, items related to content clarity and relevance received high ratings from the expert judges, indicating conceptual and terminological consistency ^(26)^. A study that developed and validated an instrument to support information transfer in the care of preterm newborns found that rigorous validation methods yield tools that are methodologically sound and conceptually straightforward, key elements for standardizing communication and promoting patient safety in pediatric settings ^(27)^. The predominance of judges with extensive experience in nursing and pediatric care further strengthens the panel, helping ensure that assessments and recommendations align with the needs of the clinical context. Accordingly, including experts with substantial field experience is an important factor for rigorous appraisal and for aligning instruments with clinical and scientific best practices ^(28)^.

Expert involvement in instrument validation supports clinical applicability and helps ensure item clarity and representativeness, which can increase acceptance and adoption among professionals ^(29-31)^. However, the literature emphasizes that handoff tools must be well structured, as ineffective communication among health care professionals is associated with adverse events and compromised patient safety ^(32)^.

In this study, the domains “Clarity,” “Practical relevance,” and “Appropriateness to the PICU context” received high scores, reflecting consensus among the judges regarding the items’ relevance. However, effective use of this tool depends on a sufficiently prepared environment, professionals trained to use it, and efforts to minimize communication distractions, thereby strengthening patient safety during handoffs ^(33)^. Recent research indicates that staff training and team adaptation are key determinants of the tool’s effectiveness, underscoring the need for ongoing training programs ^(34)^. Building SBAR competency among professionals remains challenging. Although SBAR is widely recognized for structuring communication clearly and objectively, implementation continues to face barriers related to clinicians’ familiarity with the method and integration into institutional routines ^(22,32)^. Despite these implementation challenges, SBAR continues to be cited as an effective strategy for organizing information exchanged across shifts and hospital units, promoting greater standardization and safer care delivery ^(35)^.

Accordingly, the present study reaffirms the importance of a standardized instrument for care handoffs in pediatric units. Evidence from content and face validation, together with semantic analysis, suggests that the instrument developed is suitable for clinical use, supporting patient safety and the quality of nursing care. Its implementation can help standardize handoff communication processes in pediatric units. It is important to note that the domains with the highest agreement were those addressing item relevance and applicability in practice. Items with lower agreement scores indicate a need for terminology refinement and more detailed specification of the proposed actions, as reflected in the expert judges’ suggestions.

### Study limitations

Validation was limited to evidence from content and face validation, along with semantic analysis; the instrument was not applied in real-world PICU care settings. This fact limits the generalizability of the findings and precludes inferences about performance during routine use ^(36)^. In addition, some expert judges showed limited familiarity with SBAR, reflected in low participation in prior training, which may have influenced their assessment of item clarity and representativeness ^(31)^. Finally, although the panel was qualified, the number of judges may limit the extrapolation of the results to other contexts.

### Contributions to the field of health, nursing, and public policy

This study contributes to the standardization of communication in Pediatric Intensive Care through a validated instrument that enhances patient safety and continuity of care. The tool supports clinical decision-making and qualifies nursing practice, continuing education, and care management.

## CONCLUSIONS

The study enabled the construction and validation of an SBAR-based instrument for transition of care in the Pediatric Intensive Care Unit. The theoretical foundation through scoping review and validation by 15 experts ensured methodological rigor, with Kappa and CVI values exceeding 0.70. Semantic analysis confirmed the clarity and applicability of the tool with the target audience. The implementation of this checklist aims to standardize interprofessional communication and strengthen patient safety. Future studies are suggested to evaluate the direct clinical impact of the tool in different hospital settings. Initial implementation is intended for a federal university hospital, following training of the nursing team.

## Data Availability

The data and materials used in this study are available from the corresponding author upon request.
