# Supplementary material for: SBAR checklist for the transition of care in nursing in the Pediatric Intensive Care Unit
Source: Rev Bras Enferm. 2026 Oct 19;79:e20250398. doi: 10.1590/0034-7167-2025-0398 (PMC13679586; doi:10.1590/0034-7167-2025-0398)
Supplement: Translation

# Translation: Checklist SBAR para a transição do cuidado em enfermagem na Unidade de Terapia Intensiva Pediátrica

**DOI:** 10.1590/0034-7167-2025-0398pt

**Authors:** Thales Henrique Santos, Juliana Teixeira Jales Menescal Pinto, Micheline Veras de Moura, Izaura Luzia Silvério Freire, Fernanda Maria Wanderley Cavalcanti Freire, José Segundo Barbosa, Sandra Ávila Cavalcante, Jayana Teixeira Jales Menescal Pinto

**Affiliations:** I Universidade Federal do Rio Grande do Norte. Natal, Rio Grande do Norte, Brasil; II Universidade do Porto, Escola Superior de Enfermagem do Porto. Porto, Portugal

**Keywords:** Comunicação, Cuidado Transicional, Enfermagem Pediátrica, Unidades de Terapia Intensiva Pediátrica, Pesquisa Metodológica em Enfermagem.

## Abstract

**Objetivo:**

Construir e validar um instrumento baseado no mnemônico SBAR para a transição do cuidado em Unidades de Terapia Intensiva Pediátrica.

**Método:**

Estudo metodológico fundamentado no referencial de Pasquali, desenvolvido em três polos (teórico, empírico e analítico). Os itens foram elaborados via revisão de escopo (PRISMA-ScR). O instrumento foi submetido à validação de conteúdo e aparência por 15 especialistas e à análise semântica por três residentes de enfermagem. A concordância foi estimada pelo IVC e coeficiente Kappa (>0,70).

**Resultados:**

A versão final consolidou-se em cinco domínios (Identificação, Situação, Breve Histórico, Avaliação e Recomendações) e 41 itens. Todos apresentaram IVC e Kappa superiores a 0,70. A análise semântica confirmou a compreensão adequada sem necessidade de ajustes.

**Conclusão:**

O instrumento mostrou-se válido e aplicável, constituindo uma tecnologia assistencial capaz de padronizar a comunicação e elevar a segurança do paciente pediátrico durante a transferência de informações.

## INTRODUÇÃO

A comunicação constitui a base do cuidado em saúde, sendo um processo dinâmico que envolve linguagem verbal e não verbal, como expressões faciais, gestos e postura ^(1)^. Ao longo dos anos, o conceito de comunicação evoluiu de uma simples transmissão de informações para um processo complexo e interativo, influenciado pelos avanços tecnológicos e pelas exigências crescentes de qualidade e segurança na assistência ^(2)^. No contexto hospitalar, garantir uma comunicação eficaz entre os profissionais de saúde é um desafio crítico para a promoção da segurança do paciente, fortalecimento dos vínculos com os usuários e prevenção de incidentes ^(1)^.

O Ministério da Saúde do Brasil reconhece essa importância ao estabelecer dispositivos normativos como a Portaria nº 1.820/2009, que assegura aos usuários o direito à informação clara e compreensível sobre seu estado de saúde, e a Portaria nº 529/2013, que institui o Programa Nacional de Segurança do Paciente (PNSP), cujas metas incluem a “melhoria da comunicação entre os profissionais de saúde” ^(3)^.

Diversos estudos nacionais e internacionais apontam a comunicação ineficaz como fator contribuinte para a ocorrência de eventos adversos (EA), os quais podem causar danos físicos e emocionais aos pacientes, prolongar hospitalizações e aumentar os custos dos serviços de saúde ^(4)^. Uma pesquisa em hospitais dinamarqueses revelou que 86% das falhas de comunicação ocorreram durante a transição do cuidado, momento crítico em que informações devem ser repassadas de forma clara e completa entre turnos, setores ou instituições ^(4)^. Na Unidade de Terapia Intensiva Pediátrica, a comunicação torna-se ainda mais sensível, considerando a vulnerabilidade clínica das crianças e a necessidade de constante articulação entre a equipe multiprofissional e os familiares. A transição do cuidado (TC), entendida como a transferência de responsabilidade e de informações sobre o cuidado do paciente, deve ocorrer de maneira estruturada, clara e segura para evitar lacunas assistenciais, especialmente em contextos de alta complexidade ^(5,6)^.

Dessa perspectiva, destaca-se o mnemônico SBAR (*Situation, Background, Assessment, Recommendation*), ferramenta estruturada que organiza a comunicação clínica com base em quatro componentes essenciais, favorecendo a transmissão de informações de forma objetiva, padronizada e segura.

Reconhecido por instituições internacionais como o Institute for Healthcare Improvement (IHI), o SBAR tem sido cada vez mais incorporado às práticas de enfermagem como tecnologia assistencial, especialmente em unidades críticas como a Unidade de Terapia Intensiva Pediátrica, onde erros de comunicação podem comprometer seriamente a segurança do cuidado ^(7)^. A prática assistencial de enfermagem em uma Unidade de Terapia Intensiva Pediátrica de hospital universitário permitiu identificar falhas recorrentes na comunicação, principalmente durante as passagens de plantão, as transferências de cuidado, os registros no prontuário e as prescrições médicas. Tais falhas colocam em risco a continuidade do cuidado, a segurança da criança e a qualidade do serviço prestado. O cenário observacional, aliado à escassez de instrumentos padronizados para a transição do cuidado pediátrico baseados no SBAR, evidenciou uma lacuna importante na literatura e na prática clínica, reafirmando a necessidade de desenvolver e validar tecnologias que sistematizem a comunicação e reduzam os riscos. Diante do exposto, este estudo tem como objetivo construir e validar um instrumento baseado no mnemônico SBAR e direcionado à transição do cuidado de crianças e adolescentes em uma Unidade de Terapia Intensiva Pediátrica, com vistas à qualificação da comunicação entre os profissionais de saúde, à segurança do paciente e à efetividade do cuidado de enfermagem.

**Relevância do estudo:** Este artigo é produto da Dissertação^(8)^ intitulada “Construção e validação de instrumento para a transição do cuidado de crianças e adolescentes de Unidade de Terapia Intensiva Pediátrica”, depositada no repositório institucional da Universidade Federal do Rio Grande do Norte (UFRN) e disponível em: https://repositorio.ufrn.br/items/db82a1fa-502d-49ca-9ce8-2bb40527e39e.

## OBJETIVO

Construir e validar um instrumento baseado no mnemônico SBAR, voltado para a transição do cuidado de crianças e adolescentes em uma Unidade de Terapia Intensiva Pediátrica, visando à qualificação da comunicação entre os profissionais de saúde, à segurança do paciente e à efetividade do cuidado de enfermagem.

## MÉTODOS

### Aspectos éticos

O estudo foi conduzido conforme as diretrizes éticas nacionais e internacionais e aprovado pelo Comitê de Ética da Universidade Federal do Rio Grande do Norte (UFRN).

### Desenho, período e local do estudo

Trata-se de um estudo original, de natureza metodológica e de abordagem quantitativa. Seguiu as diretrizes metodológicas recomendadas para pesquisas de construção e validação de instrumentos, de acordo com o checklist *COnsensus-based Standards for the selection of health Measurement INstruments* (COSMIN), da rede EQUATOR e PRISMA-ScR para a etapa de revisão ^(9,10)^. Para garantir a transparência e reprodutibilidade do processo, uma revisão de escopo foi realizada como etapa inicial para fundamentar a construção do instrumento, seguindo as recomendações PRISMA-ScR (Scoping Reviews). Seu protocolo foi previamente registrado na plataforma Open Science Framework (OSF) (DOI: 10.17605/OSF.IO/D93JM) e está disponível em acesso aberto no seguinte link: https://osf.io/d93jm/. O estudo foi conduzido entre 2023 e 2025, na cidade de Natal, estado do Rio Grande do Norte, Brasil.

### População ou amostra; critérios de inclusão e exclusão

A amostra para a fase de validação teórica do instrumento foi composta por 15 juízes especialistas, selecionados intencionalmente, incluindo enfermeiros especialistas em enfermagem pediátrica e docentes com experiência em pesquisa metodológica e pediatria. Foram incluídos especialistas com comprovada experiência e atuação na área. Juízes em férias, afastamento ou licença foram excluídos.

### Protocolo do estudo

O estudo foi desenvolvido em três polos principais, conforme o referencial de Pasquali: Polo 1 - Procedimentos Teóricos (Fundamentação e Construção): Realização de revisão de escopo para identificar os itens relevantes e elaboração do instrumento piloto estruturado em cinco domínios baseados no SBAR. Polo 2 - Procedimentos Empíricos (Validação e Semântica): Avaliação do instrumento por 15 juízes especialistas para validação de conteúdo e aparência, além da análise semântica por meio da técnica de *brainstorming* com residentes de enfermagem. Polo 3 - Procedimentos Analíticos (Análise Estatística): Processamento e análise dos dados coletados para verificação dos índices de concordância (IVC e Kappa), garantindo a fidedignidade do instrumento final de 41 itens.

### Análise dos resultados e estatística

Os dados foram organizados em planilhas e analisados por estatística descritiva. A concordância entre os juízes foi mensurada pelo Índice de Validade de Conteúdo (IVC) e pelo coeficiente de Kappa (k). Adotou-se o ponto de corte de > 0,70 para considerar o item válido e compreensível.

## RESULTADOS

O instrumento, apoiado em revisão de escopo, compreendeu cinco domínios essenciais: identidade do paciente; situação atual; histórico clínico; e avaliação do estado dos sistemas e recomendações para continuidade do cuidado. A primeira versão, composta por 38 itens, foi avaliada por 15 especialistas, cujas sugestões foram incorporadas para aprimorar a clareza e a abrangência do instrumento^(8,11)^.

O painel avaliador foi composto predominantemente por mulheres (93,33%), com formação em especialização (46,67%) e mestrado (40%). A maioria dos especialistas eram pardos (60%), católicos (66,67%) e residentes em Natal (86,67%). Apesar de 86,67% não possuírem treinamento prévio específico em SBAR, isso não comprometeu a validação focada na relevância e representatividade dos itens. A média de idade foi 40,67 anos (DP = 7,45), com ampla experiência em enfermagem (16,8 anos) e em pediatria (11,07 anos). Assim, a validação consistiu na avaliação que profissionais experientes fizeram dos indicadores oriundos da literatura para a transição do cuidado mediada por comunicação estruturada. A Tabela 1 mostra o perfil dos avaliadores que participaram da validação do instrumento.

**Tabela 1:** Perfil dos avaliadores que participaram da validação do instrumento. Natal, Rio Grande do Norte, Brasil, 2024

Caracterização	Frequência absoluta	%
**Sexo**		
Feminino	14	93,33
Masculino	1	6,67
**Escolaridade**		
Especialização/residência	7	46,67
Mestrado	6	40,00
Doutorado	2	13,33
**Etnia**		
Pardo	9	60,00
Branco	6	40,00
**Religião**		
Católica	10	66,67
Evangélica	3	20,00
Sem crença	1	6,67
Outros	1	6,67
**Estado civil**		
Casado/união estável	10	66,67
Solteiro	4	26,67
Divorciado	1	6,67
**Procedência**		
Natal	13	86,67
Mossoró	1	6,67
Parnamirim	1	6,67
**Treinamento relacionado ao SBAR**		
Sim	2	13,33
Não	13	86,67
**Total**	15	100,00

A avaliação feita pelos especialistas resultou em modificações nos domínios do instrumento, com o objetivo de aprimorar sua clareza e aplicabilidade clínica. No domínio “Identificação”, a linguagem foi ajustada para maior objetividade. No domínio “Situação”, os itens foram simplificados para facilitar o preenchimento rápido. Os domínios “Breve histórico” e “Avaliação” foram revisados para incorporar indicadores clínicos específicos da pediatria, excluindo itens redundantes. Por fim, o domínio “Recomendações” foi ajustado para garantir a continuidade do cuidado de forma segura. A Tabela 2 detalha as alterações quantitativas em cada domínio.

**Tabela 2:** Alterações nos itens do instrumento por domínio. Natal, Rio Grande do Norte, Brasil, 2024

Domínio	Itens originais	Itens excluídos	Itens reformulados	Itens incluídos	Itens finais
1. Identificação	8	0	2	2	10
2. Situação	10	1	4	2	11
3. Breve histórico	7	1	3	2	8
4. Avaliação	9	2	4	2	9
5. Recomendações	4	1	2	0	3
Total	38	5	15	8	41

Observa-se que a versão final consolidou-se em 41 itens, resultado da inclusão de novas questões consideradas essenciais, equilibrando a exclusão de itens redundantes. Os Índices de Validade de Conteúdo (IVC) foram calculados para cada item, evidenciando elevada concordância entre os avaliadores. A Tabela 3 apresenta o IVC por domínio.

**Tabela 3:** Índices de Validade de Conteúdo por domínio. Natal, Rio Grande do Norte, Brasil, 2024

Domínio	Número de itens finais	IVC médio	Kappa médio
Identificação	10	1,00	1,00
Situação	11	1,00	1,00
Breve histórico	8	0,93	0,73
Avaliação	9	1,00	1,00
Recomendações	3	1,00	1,00

Após as revisões sugeridas, os valores indicam que o instrumento é válido para aplicação clínica. Além das alterações nos itens, os especialistas sugeriram melhorias no formato para otimizar a experiência do usuário, como o uso de papel resistente, fonte Arial tamanho 12 e divisões claras entre os domínios. Essas recomendações visam garantir que o instrumento seja prático no ambiente hospitalar. O Quadro 1 apresenta as recomendações gerais de formato.

**Quadro 1:** Recomendações gerais para o formato do instrumento. Natal, Rio Grande do Norte, Brasil, 2024

Aspecto	Recomendações	Justificativa
Tipo de Papel	Papel opaco, resistente	Melhor durabilidade e manuseio
Tamanho da Página	A4	Facilita a impressão e arquivamento
Fonte	Arial, tamanho 12	Leitura confortável
Organização	Divisão clara em domínios	Facilita a navegação pelo instrumento

O instrumento finalizado contém 41 itens distribuídos em cinco domínios. A Figura 1 exibe a versão final validada.

**Figura 1:** Versão final do instrumento (frente). Natal, Rio Grande do Norte, Brasil, 2024

## DISCUSSÃO

A comunicação eficaz durante a passagem de plantão é fundamental para a segurança do paciente em Unidades de Terapia Intensiva Pediátrica. Estudos têm evidenciado que falhas nesse processo podem resultar em erros médicos e eventos adversos. As interrupções frequentes, ruídos de equipamentos e conversas paralelas são fatores que comprometem a transmissão adequada de informações entre profissionais de saúde ^(12)^. Além disso, a ausência de padronização na comunicação durante as trocas de turno pode levar a omissões ou distorções de informações significativas sobre o estado clínico dos pacientes. A implementação de ferramentas estruturadas, como o método SBAR, tem sido sugerida para melhorar a clareza e a concisão das informações transmitidas, reduzindo, assim, a ocorrência de erros ^(13)^. Tais aspectos justificam a necessidade de validação de instrumentos específicos que organizem e padronizem essas informações no contexto pediátrico, promovendo uma comunicação segura e efetiva entre os membros da equipe de saúde.

A conscientização sobre essas dificuldades e a busca por estratégias que aprimorem a comunicação na passagem de plantão foram motivadoras para a realização deste estudo, no intuito de contribuir para a melhoria da qualidade assistencial em Unidade de Terapia Intensiva Pediátrica. Esses benefícios ocorrem, principalmente, pela padronização da linguagem e pela sistematização das informações clínicas, fatores que reduzem ruídos e aumentam a eficiência da tomada de decisão ^(14,15)^.

Em relação ao Domínio 1 sobre Identificação: No domínio de Identificação, a principal alteração foi a inclusão do sexo do paciente e do grau de parentesco do acompanhante. Essa mudança aprimora a precisão dos registros e auxilia na individualização do cuidado prestado, uma vez que o grau de parentesco pode influenciar diretamente o suporte e a adesão às condutas recomendadas pela equipe de saúde ^(16)^. Registros completos e precisos são essenciais para a continuidade e segurança da assistência.

Além disso, a identificação clara do acompanhante favorece a responsabilização e o envolvimento ativo da família no processo terapêutico, aspectos especialmente relevantes no contexto pediátrico ^(16,17)^. Quanto ao Domínio 2 sobre Situação: Neste domínio, as sugestões aceitas incluíram o refinamento da descrição do estado geral, com sua categorização em “regular”, “grave” ou “comprometido”, oferecendo uma visão mais objetiva e padronizada do estado clínico do paciente. Também foram incluídas categorias mais abrangentes para o nível de consciência (incluindo “sedado” e “comatoso”) e parâmetros específicos para crianças pequenas, favorecendo uma avaliação mais adequada em diferentes faixas etárias. No item “Pele”, acrescentaram-se as opções “hidratada/desidratada” e “hiperemiada”, bem como um espaço para o registro de lesões.

Em conjunto, essas revisões sustentam uma avaliação mais padronizada e facilitam a comunicação entre os profissionais. Estudos destacam que a padronização da comunicação de informações clínicas é fundamental para a segurança do paciente ^(18)^. Nesse sentido, a incorporação de descritores objetivos favorece uma linguagem comum entre os profissionais e diminui a subjetividade nas interpretações clínicas, o que se mostra crucial em ambientes de alta complexidade como a Unidade de Terapia Intensiva Pediátrica^(19)^.

Em se tratando do Domínio 3 sobre Breve Histórico: A reformulação das comorbidades eliminou a lista fixa de patologias e incorporou categorias mais amplas (p.ex., nefropatias, pneumopatias e doenças metabólicas), com um espaço para especificação. Acrescentou-se também a opção de registrar alergias e prematuridade. Essas mudanças aumentam a flexibilidade do instrumento e garantem que informações relevantes para o cuidado sejam registradas adequadamente. Assim, elas reforçam que a comunicação eficaz dessas informações é essencial para a continuidade do cuidado e prevenção de eventos adversos ^(20)^. As referidas alterações estão em consonância com a literatura recente, que recomenda instrumentos clínicos mais dinâmicos, adaptando-se às diferentes condições de saúde e à singularidade de cada paciente ^(21,22)^.

Relativamente ao Domínio 4 sobre Avaliação: As principais modificações nesta seção incluíram a revisão do item “Sinais vitais”, destacando a relevância do teste de glicemia capilar (hemoglicoteste; HGT) para determinados pacientes. No suporte ventilatório, foi acrescentado um campo para registrar o modo ventilatório, além da FiO_2_, para melhor documentação dos parâmetros da ventilação mecânica. O item “Dispositivos” foi expandido para incluir detalhes sobre coberturas, curativos e características dos acessos venosos centrais, tornando o instrumento mais completo para a equipe assistencial. Sabe-se que comunicar efetivamente essas informações é crucial para a segurança do paciente, especialmente em unidades críticas ^(23)^.

A presença de dados específicos sobre ventilação mecânica e dispositivos reduz riscos relacionados a falhas de continuidade no cuidado e potencializa a capacidade de resposta da equipe diante de mudanças clínicas ^(15)^. Sobre o Domínio 5: Recomendações: Neste domínio, a estrutura de organização dos dados foi alterada, consolidando os itens “Procedimentos invasivos programados” e “Cirurgia solicitada/agendada”, além da reformulação do layout do item “Exames solicitados”, a fim de tornar o registro mais claro. Também foi acrescentado um espaço para registrar o transporte utilizado na alta ou transferência externa, bem como a inclusão de opções para pareceres pendentes e psicologia no item “Parecer solicitado”, favorecendo a continuidade e segurança do cuidado. A implementação de ferramentas padronizadas de comunicação, como o SBAR, tem mostrado melhorias significativas na qualidade das transferências e na delineação de responsabilidades no cuidado. Tais modificações permitem visualizar de forma integrada as ações futuras, facilitando o planejamento do cuidado por toda a equipe interdisciplinar ^(24)^.

A implementação dessas alterações reforça a eficiência do método SBAR na comunicação interprofissional. A padronização da transferência de informações permite uma passagem de plantão mais segura, reduz erros e garante que todos os aspectos relevantes do cuidado sejam considerados. Dessa forma, o aprimoramento do instrumento fortalece a segurança do paciente e a qualidade da assistência prestada na UTI pediátrica. A validação de instrumentos clínicos é um processo essencial para garantir a confiabilidade e aplicabilidade de ferramentas utilizadas na assistência em saúde ^(25)^. Estudos recentes corroboram a eficácia da validação de conteúdo e aparência, juntamente com a análise semântica, na construção de instrumentos para a prática clínica. Em pediatria, estudos de validação de instrumentos apontam uma crescente produção científica voltada a métodos robustos, com potencial para transformar a prática assistencial por meio de inovações metodológicas no processo de enfermagem. Na presente validação, itens relacionados à clareza e pertinência dos conteúdos foram bem avaliados pelos juízes, demonstrando consistência conceitual e terminológica ^(26)^.

Estudo que desenvolveu e validou um instrumento para a transição de informações no cuidado ao neonato prematuro mostrou que a aplicação de métodos rigorosos de validação resulta em ferramentas metodologicamente adequadas e conceitualmente claras, essenciais para a padronização da comunicação e promoção da segurança do paciente em unidades pediátricas ^(27)^. A predominância de especialistas com experiência consolidada em enfermagem e assistência pediátrica reforça a solidez do painel, assegurando que as avaliações e sugestões estejam em consonância com as demandas do contexto assistencial. Sob essa perspectiva, percebe-se que a inclusão de especialistas com ampla experiência na área é um fator relevante para a análise criteriosa e alinhamento dos instrumentos às boas práticas clínicas e científicas ^(28)^.

A participação de especialistas na validação de instrumentos contribui para sua aplicabilidade na prática clínica bem como garantia da clareza e representatividade dos itens, aumentando a aceitação e adesão pelos profissionais ^(29-31)^. Contudo, a literatura destaca que os instrumentos devem estar bem estruturados para a transição de cuidados, uma vez que a comunicação ineficaz entre profissionais de saúde está associada a eventos adversos e ao comprometimento da segurança do paciente ^(32)^.

Neste estudo, os domínios “Clareza”, “Relevância prática” e “Adequação à realidade da Unidade de Terapia Intensiva Pediátrica” apresentaram escores elevados, refletindo consenso entre os juízes quanto à pertinência dos itens. Entretanto, a efetiva utilização dessa ferramenta exige um ambiente devidamente preparado, profissionais capacitados para seu uso e minimização dos ruídos na comunicação, a fim de fortalecer a segurança do paciente durante a transição do cuidado ^(33)^. Pesquisas recentes evidenciam que o treinamento e a adaptação da equipe são determinantes para a efetividade dessa ferramenta, o que reforça a necessidade de programas de capacitação contínuos ^(34)^. A capacitação dos profissionais para o uso do SBAR ainda é um desafio. A literatura demonstra que, mesmo sendo um método amplamente reconhecido por sua capacidade de organizar a comunicação de forma clara e objetiva, sua implementação ainda enfrenta barreiras relacionadas à familiaridade dos profissionais e à adequação às rotinas institucionais ^(22,32)^. Mesmo diante desses desafios para sua adoção, o SBAR continua sendo apontado como uma estratégia eficaz para organizar as informações transmitidas entre turnos e unidades hospitalares, promovendo maior padronização e segurança assistencial ^(35)^.

Dessa forma, o presente estudo reafirma a importância de um instrumento padronizado para a transição de cuidado em unidades pediátricas. A validação do conteúdo e da aparência, somada à análise semântica, evidencia que o instrumento desenvolvido é adequado para ser utilizado na prática clínica, contribuindo para a segurança do paciente e qualidade da assistência de enfermagem. Sua implementação favorecerá a padronização do processo de comunicação na transição do cuidado em unidades pediátricas. É importante destacar que os domínios com maior concordância foram os que abordaram a pertinência dos itens e sua aplicabilidade no cenário prático. Os itens com menor índice de concordância apontam para a necessidade de ajustes na terminologia utilizada e no detalhamento das ações propostas, conforme observado nas sugestões dos juízes.

### Limitações do estudo

A validação foi restrita às evidências de validade de conteúdo e aparência, além da análise semântica, sem aplicação do instrumento em cenários reais de assistência na Unidade de Terapia Intensiva Pediátrica, o que limita a generalização dos achados e impede inferências sobre seu desempenho em uso ^(36)^. Ademais, observou-se baixa familiaridade de parte dos especialistas com o método SBAR, evidenciada pela participação reduzida em treinamentos prévios, o que pode ter influenciado a avaliação da clareza e representatividade dos itens ^(31)^.

### Contribuições para a área da Enfermagem, Saúde ou Política Pública

Este estudo contribui para a padronização da comunicação em Terapia Intensiva Pediátrica por meio de um instrumento validado que potencializa a segurança do paciente e a continuidade do cuidado. A ferramenta subsidia decisões clínicas e qualifica a prática de enfermagem, a educação permanente e a gestão assistencial.

## CONCLUSÕES

O estudo possibilitou a construção e validação de um instrumento baseado no mnemônico SBAR para a transição do cuidado em Unidade de Terapia Intensiva Pediátrica. A fundamentação teórica por revisão de escopo e a validação por 15 especialistas garantiram o rigor metodológico, com índices de Kappa e IVC superiores a 0,70. A análise semântica confirmou a clareza e a aplicabilidade da ferramenta junto ao público-alvo. A implementação deste checklist visa padronizar a comunicação interprofissional e fortalecer a segurança do paciente. Sugerem-se estudos futuros para avaliar o impacto clínico direto da ferramenta em diferentes cenários hospitalares. Pretende-se, inicialmente, aplicar o instrumento em um hospital universitário federal, mediante capacitação da equipe de enfermagem.

## Data Availability

Os dados e materiais utilizados nesta pesquisa estão disponíveis mediante solicitação ao autor correspondente.
